# Male histone deacetylase 6 (HDAC6) knockout mice have enhanced ventilatory responses to hypoxic challenge

**DOI:** 10.3389/fphys.2023.1332810

**Published:** 2024-02-06

**Authors:** Paulina M. Getsy, Gregory A. Coffee, Thomas J. Kelley, Stephen J. Lewis

**Affiliations:** ^1^ Department of Pediatrics, Case Western Reserve University, Cleveland, OH, United States; ^2^ Department of Genetics and Genome Sciences, CWRU, Cleveland, OH, United States; ^3^ Department of Pharmacology, CWRU, Cleveland, OH, United States; ^4^ Functional Electrical Stimulation Center, CWRU, Cleveland, OH, United States

**Keywords:** hypoxia, histone deacetylase 6 (HDAC6), mice, HIF-1α, whole body plesthysmography

## Abstract

Histone deacetylase 6 (HDAC6) is a class II histone deacetylase that is predominantly localized in the cytoplasm of cells. HDAC6 associates with microtubules and regulates acetylation of tubulin and other proteins. The possibility that HDAC6 participates in hypoxic signaling is supported by evidence that 1) hypoxic gas challenges cause microtubule depolymerization, 2) expression of hypoxia inducible factor alpha (HIF-1α) is regulated by microtubule alterations in response to hypoxia, and 3) inhibition of HDAC6 prevents HIF-1α expression and protects tissue from hypoxic/ischemic insults. The aim of this study was to address whether the absence of HDAC6 alters ventilatory responses during and/or after hypoxic gas challenge (10% O_2_, 90% N_2_ for 15 min) in adult male wildtype (WT) C57BL/6 mice and HDAC6 knock-out (KO) mice. Key findings were that 1) baseline values for frequency of breathing, tidal volume, inspiratory and expiratory times, and end expiratory pause were different between knock-out mice and wildtype mice, 2) ventilatory responses during hypoxic challenge were more robust in KO mice than WT mice for recorded parameters including, frequency of breathing, minute ventilation, inspiratory and expiratory durations, peak inspiratory and expiratory flows, and inspiratory and expiratory drives, and 3) responses upon return to room-air were markedly different in KO compared to WT mice for frequency of breathing, minute ventilation, inspiratory and expiratory durations, end expiratory pause (but not end inspiratory pause), peak inspiratory and expiratory flows, and inspiratory and expiratory drives. These data suggest that HDAC6 may have a fundamentally important role in regulating the hypoxic ventilatory response in mice.

## Highlights

• Resting ventilatory parameters, such as frequency of breathing, tidal volume, inspiratory and expiratory times, and end expiratory pause, were different between male histone deacetylase 6 (HDAC6) knock-out (KO) mice and wildtype (WT) C57BL/6 mice.
• HDAC6 KO mice have enhanced hypoxic ventilatory responses compared to WT.
• Ventilatory parameters recorded after hypoxic gas challenge (i.e., upon return to room-air) are markedly different in HDAC6 KO mice compared to WT.
• HDAC6 may have a fundamentally important role in regulating the response to hypoxia in mice.

## Introduction

Histone deacetylase 6 (HDAC6) is a class II histone deacetylase that exists predominantly within the cytosolic compartment of cells where it associates with microtubules to regulate the acetylation of tubulin and other cytosolic/intracellular protein targets (Li et al., 2011; Seidel et al., 2015; Rodrigues et al., 2016; Balmik and Chinnathamb, 2022; Bonanni et al., 2022; Xu and Wan, 2023). Numerous studies have demonstrated that pharmacological inhibition of HDAC6 improves neuronal function in a variety of disease states (Li et al., 2011; Seidel et al., 2015; Rodrigues et al., 2016; Liang and Fang, 2018; Ma et al., 2018; Prior et al., 2018; LoPresti, 2020; Kumar et al., 2022; Qureshi and Chinnathambi, 2022). For example, inhibition of HDAC6 improves microtubule-mediated transport in neurons in Huntington’s disease directly by increasing tubulin acetylation (Dompierre et al., 2007). The peripheral nerve disease, Charcot-Marie-Tooth, is characterized by reduced tubulin acetylation (Ydewalle et al., 2011). HDAC6 inhibitors improve neuronal transmission and alleviate phenotypes in a mouse model of this disease (Ydewalle et al., 2011; Benoy et al., 2018). In addition, HDAC6 inhibitors have been assessed in vascular dementia (e.g., Alzheimer’s disease) and Parkinson’s disease based on their ability to improve neuronal function *via* tubulin acetylation (Godena et al., 2014; Majid et al., 2015; Cuadrado-Tejedor et al., 2017; Esteves et al., 2018).

Clear relationships between hypoxia and microtubule regulation have been demonstrated in cardiomyocyte preparations (Teng et al., 2010; Dang et al., 2015). For example, Dang et al. (2015) demonstrated that hypoxic (**HX**) gas challenge leads to microtubule depolymerization. These findings are consistent with those from Teng et al. (2010) who demonstrated that hypoxia inducible factor-1 alpha (HIF-1α) expression is regulated by microtubule alterations in response to HX gas challenge. Stable microtubules are preferentially acetylated, suggesting that inhibition of HDAC6 may protect against damage induced by HX insults. Inhibition of HDAC6 prevents HIF-1α expression and protects tissues from injury resulting from HX or ischemic challenges (Kong et al., 2006; Qian et al., 2006; Su et al., 2016; Leng et al., 2018). Taken together, it is evident that the pharmacological inhibition of HDAC6 protects against hypoxic challenge-induced tissue damage, and also improves central and peripheral neuronal function in numerous disease states (Kong et al., 2006; Qian et al., 2006; Dompierre et al., 2007; Teng et al., 2010; Li et al., 2011; Ydewalle et al., 2011; Godena et al., 2014; Dang et al., 2015; Majid et al., 2015; Seidel et al., 2015; Rodrigues et al., 2016; Su et al., 2016; Cuadrado-Tejedor et al., 2017; Benoy et al., 2018; Esteves et al., 2018; Leng et al., 2018; Liang and Fang, 2018; Ma et al., 2018; Prior et al., 2018; LoPresti, 2020; Balmik and Chinnathamb, 2022; Bonanni et al., 2022; Kumar et al., 2022; Qureshi and Chinnathambi, 2022; Xu and Wan, 2023).

Ventilatory responses to HX gas challenges are dependent on carotid body sensing and neuronal (chemoreceptor afferent) signal propagation to the commissural nucleus tractus solitarius in the brainstem (Lahiri et al., 2006; Prabhakar and Peers, 2014; López-Barneo et al., 2016; Baby et al., 2018). At present, there is no information as to whether HDAC6 exists in primary glomus (hypoxia-sensing) cells within the carotid body, or in key brain structures, such as the commissural nucleus tractus solitarius, that receive and process chemoreceptor afferent input. The evidence that HDAC6 inhibition prevents HIF-1α expression and protects tissue from HX and/or ischemic damage (Qian et al., 2006; Su et al., 2016; Tsai et al., 2016; Yi et al., 2019), suggests a role for HDAC6 in carotid body function since there is extensive evidence that HIF-1α has many roles in HX signaling in primary carotid body glomus cells (Baby et al., 2003; Baby et al., 2004; Roy et al., 2004a; Roy et al., 2004b; Roy et al., 2007; Lahiri et al., 2009).

To our knowledge, there are no studies that have directly addressed whether HDAC6 has a role in signaling processes involved in the ventilatory responses that occur upon exposure to HX gas challenge. As such, the objective of this study was to compare the ventilatory responses elicited by a HX gas challenge (10% O_2_, 90% N_2_) in adult male wildtype (WT) C57BL/6 mice and HDAC6 knock-out (KO) mice by whole body plethysmography (Palmer et al., 2013a; Palmer et al., 2013b; Palmer et al., 2015; Gaston et al., 2014; Getsy et al., 2014; Getsy et al., 2023a; Getsy et al., 2023b). The data from these studies demonstrate that HDAC6 has an important role in regulating the neural responses that drive the ventilatory responses to HX gas challenge.

## Experimental procedures

All studies described were carried out in accordance with the National Institutes of Health Guide for the Care and Use of Laboratory Animals (NIH Publication No. 80-23) revised in 1996. The protocols were approved by the Animal Care and Use Committee of Case Western Reserve University.

### Mice

Adult male and female HDAC6 KO (Hdac6^−/−^) mice were generously provided by Dr. Tso-Pang Yao (Duke University). Breeding pairs of these mice provided the adult male HDAC6 KO mice used in this study. As shown in Supplementary Figure S1, using specific primers to amplify the HDAC6 gene region where the KO is located, we globally identified the KO strain as well as the heterozygous mice. We have also functionally characterized this line of KO mice for an increase in acetylated-alpha-tubulin content consistent with a loss of HDAC6 expression (Rymut et al., 2017).

### Whole body plethysmography

Ventilatory parameters in freely-moving male mice were recorded by whole body plethysmography (PLY3223; *Data Sciences International*, St. Paul, MN) as described previously (Palmer et al., 2013a; b, 2015; Gaston et al., 2014; Getsy et al., 2014; Getsy et al., 2002; Getsy et al., 2023a; Getsy et al., 2023b). The parameters recorded and derived (Supplementary Table S1; Supplementary Figure S2) were frequency of breathing (Freq); tidal volume (TV, volume of inspired air per breath); minute ventilation (Freq x TV, total volume of air inspired/min); inspiratory time (Ti, duration of inspiration); expiratory time (Te, duration of expiration); expiratory/inspiratory time (Te/Ti, expiratory quotient); end inspiratory pause (EIP, pause between end of inspiration and start of expiration); end expiratory pause (EEP, pause between end of expiration and start of inspiration); peak inspiratory flow (PIF); peak expiratory flow (PEF); airflow at 50% expired TV (EF_50_); relaxation time (RT, time to exhale 64% of TV); expiratory delay (Te-RT); inspiratory drive (TV/Ti); expiratory drive (TV/Te); non-eupneic breathing index (NEBI, % of breaths non-eupneic breaths including irregular breaths, apneas and type 1 and 2 sighs); and NEBI/Freq (NEBI corrected for Freq). The *Fine Pointe (BUXCO)* software constantly corrected digitized values for changes in chamber temperature and humidity. Pressure changes associated with the respiratory waveform were converted to volumes (e.g., TV, PIF, PEF) using the algorithm of Epstein and others (Epstein and Epstein, 1978; Epstein et al., 1980). Factoring in the chamber temperature and humidity, the cycle analyzers filtered the acquired signals, and algorithms (Fine Pointe, BUXCO) generated an array of box flow data that identified a waveform segment as an acceptable breath. From that data array, the minimum and maximum box flow values were then determined and multiplied by a compensation factor provided by the selected algorithm (Epstein and Epstein, 1978; Epstein et al., 1980), thus producing TV, PIF and PEF used to determine accepted and rejected waveforms. In the protocol described below, the conscious unrestrained mice were placed in the plethysmography chambers and allowed at least 60 min to acclimatize before exposure to the gas challenges.

### Protocols for hypoxic (HX) gas challenges

On the day of the study, C57BL/6 mice and HDAC6 KO mice were placed in whole-body plethysmography chambers and given at least 60 min to acclimatize and settle so that baseline breathing values could be ascertained. The mice were then exposed to a HX gas (10% O_2_, 90% N_2_) challenge for 5 min after which time they were re-exposed to room-air.

### Statistics

All data are shown as mean ± SEM. To determine total responses (cumulative %changes from pre-hypoxia values) during hypoxic gas challenge and total responses during return to room-air (also cumulative %changes from pre-hypoxia values) for each mouse, we summed the values recorded 1) before the hypoxic gas challenge (pre-hypoxia values), 2) during the hypoxic gas challenge and 3) upon return to room-air. We then determined the cumulative response by the formula, total response (%change) = {[(sum of values during hypoxic challenge or return to room air)—(sum of values before hypoxic challenge)]/sum of values before hypoxic challenge} x 100. We then determined the mean and SEM of the group data. All between-group data were analyzed by one-way ANOVA (Getsy et al., 2023a; Getsy et al., 2023b). Statistical analyses were performed using GraphPad Prism software (Version 9.5.1–2023; *GraphPad Software*, Inc., La Jolla, CA, United States).

## Results

### Baseline parameters

The ages of the HDAC6 KO mice were slightly lower (−3.0%) than those of WT mice, whereas the body weights of the HDAC6 KO mice were slightly higher (+13.2%) than the WT mice (Table 1). As such, the body weight/age ratio for HDAC6 KO mice (0.34 ± 0.01) was higher than that of the WT mice (0.29 ± 0.1) suggesting the possibility of the heavier body weights of the HDAC6 KO mice influencing the findings related to flow parameters, namely, TV, MV, PIF, PEF and EF_50_. Accordingly, 1) resting TV was higher in HDAC6 KO mice than in WT mice but was similar to WT when corrected for body weight; and 2) corrections for body weight did not alter the lack of differences between the two groups with respect to MV, PIF, PEF or EF_50_. Also summarized in Table 1, resting Freq was lower in the HDAC6 KO mice than in WT mice, and Ti and Te were longer in HDAC6 KO mice. In addition, resting expiratory delay (Te-RT) was longer in HDAC6 KO mice than in WT mice. All other baseline parameters were similar between the two groups. It was also evident that the moment-to moment variability of many parameters was higher in HDAC6 KO mice than in the WT mice. As shown in Table 2, values of standard deviation/corrected for mean (STDEV/mean) for Freq, MV, Ti, Te, EF_50_, PIF, PEF, EIP, expiratory time - relaxation time, and inspiratory drive were higher in HDAC6 KO mice than in WT mice. The finding that NEBI or NEBI/Freq were not different between the groups suggests that the variability is due to simple changes in breath-to-breath levels of frequency of breathing, rather than enhanced expression of non-eupneic breathing, including irregular breaths and apneas.

**TABLE 1:** Baseline parameters in wildtype (WT) and HDAC6 knockout (HDAC6 KO) mice.

Parameter	Abbreviation	WT mice	HDAC6 KO mice
Number of mice in each group		7	14
Age, (days)		94.7 ± 0.9	91.9 ± 0.6*
Body Weight (BW), (g)		27.3 ± 0.6	30.9 ± 0.9*
Body Weight/Age, (g/days)		0.29 ± 0.1	0.34 ± 0.01*
Frequency, (breaths/min)	Freq	200 ± 6	170 ± 5*
Inspiratory Time, (sec)	Ti	0.111 ± 0.003	0.133 ± 0.005*
Expiratory Time, (sec)	Te	0.210 ± 0.008	0.253 ± 0.009*
Expiratory Time/Inspiratory Time	Te/Ti	2.01 ± 0.08	1.97 ± 0.10
End Inspiratory Pause, (msec)	EIP	2.59 ± 0.09	2.68 ± 0.07
End Expiratory Pause, (msec)	EEP	30.6 ± 6.1	61.0 ± 8.5*
Tidal Volume, (mL)	TV	0.166 ± 0.010	0.200 ± 0.008*
**TV/body weight, (mL/g) x 1,000	TV/BW	6.10 ± 0.35	6.51 ± 0.26
Minute Ventilation (MV), (mL/min)	MV	32.9 ± 3.0	34.9 ± 2.5
**MV/body weight, (mL/g) x 1,000	MV/BW	1,215 ± 133	1,134 ± 79
Peak Inspiratory Flow (PIF), (mL/sec)	PIF	2.73 ± 0.38	2.72 ± 0.26
**PIF/body weight, (mL/g) x 1,000	PIF/BW	101.4 ± 16.4	88.5 ± 8.5
Peak Expiratory Flow (PEF), (mL/sec)	PEF	1.63 ± 0.15	1.77 ± 0.10
**PEF/body weight, (mL/g) x 1,000	PEF/BW	60.3 ± 6.2	57.6 ± 3.3
PEF/PIF		0.62 ± 0.04	0.71 ± 0.03
**(PEF/PIF)/body weight (ratio/g) x 1,000		22.7 ± 1.1	23.2 ± 1.1
Air-flow at 50% expired TV, (mL/sec)	EF_50_	0.079 ± 0.007	0.077 ± 0.006
**EF_50_/body weight (ratio/g) x 1,000	EF_50_/BW	2.93 ± 0.30	2.51 ± 0.21
Relaxation Time, (sec)	RT	0.104 ± 0.006	0.122 ± 0.005
Expiratory Delay	Te-RT	0.106 ± 0.005	0.128 ± 0.005*
Inspiratory Drive, (mL/sec)	TV/Ti (InspD)	1.62 ± 0.15	1.46 ± 0.10
**(TV/Ti)/body weight (ratio/g) x 1,000	InspD/BW	59.7 ± 6.4	47.5 ± 2.9
Expiratory Drive, (mL/sec)	TV/Te (ExpD)	0.80 ± 0.05	0.75 ± 0.03
**(TV/Te)/body weight (ratio/g) x 1,000	ExpD/BW	29.5 ± 2.3	24.5 ± 1.1
Non-eupneic breathing Index, (%)	NEBI	10.3 ± 1.3	13.8 ± 1.8
NEBI/Freq, [%/(breaths/min)]	NEBI/Freq	0.057 ± 0.012	0.088 ± 0.013

The data are presented as mean ± SEM. *p < 0.05, HDAC6 KO *versus* WT. **Represent the actual delta/body weight values multiplied by 1,000.

**TABLE 2:** Variability in resting parameters in wildtype (WT) and HDAC6 knockout (HDAC6 KO) mice.

Parameter	Parameter	WT mice	HDAC6 KO mice
Frequency (breaths/min)	STDEV	45.5 ± 7.4	74.7 ± 7.8*
	Mean	228 ± 20	225 ± 16
	STDEV/Mean	0.195 ± 0.019	0.325 ± 0.023*
Tidal Volume (mL)	STDEV	0.031 ± 0.003	0.038 ± 0.003
	Mean	0.170 ± 0.010	0.200 ± 0.008
	STDEV/Mean	0.180 ± 0.014	0.188 ± 0.009
Minute Ventilation (mL/min)	STDEV	12.5 ± 2.6	22.8 ± 3.0*
	Mean	39.4 ± 5.7	47.1 ± 5.1
	STDEV/Mean	0.304 ± 0.028	0.474 ± 0.036*
Inspiratory Time (sec)	STDEV	0.017 ± 0.002	0.032 ± 0.003*
	Mean	0.100 ± 0.009	0.113 ± 0.007
	STDEV/Mean	0.179 ± 0.030	0.297 ± 0.032*
Expiratory Time (sec)	STDEV	0.037 ± 0.004	0.059 ± 0.003*
	Mean	0.200 ± 0.010	0.217 ± 0.010
	STDEV/Mean	0.184 ± 0.020	0.277 ± 0.019*
Expiratory Time/Inspiratory Time	STDEV	0.457 ± 0.044	0.580 ± 0.028*
	Mean	2.122 ± 0.135	2.076 ± 0.082
	STDEV/Mean	0.218 ± 0.024	0.286 ± 0.019
End Inspiratory Pause (msec)	STDEV	0.440 ± 0.16	1.56 ± 0.68*
	Mean	2.58 ± 0.10	2.84 ± 0.14
	STDEV/Mean	0.165 ± 0.054	0.460 ± 0.149*
End Expiratory Pause (msec)	STDEV	29.7 ± 2.3	44.4 ± 4.4*
	Mean	31.0 ± 6.0	47.9 ± 4.9*
	STDEV/Mean	1.156 ± 0.209	0.954 ± 0.058
Peak Inspiratory Flow (PIF) (mL/sec)	STDEV	1.215 ± 0.227	1.895 ± 0.168
	Mean	3.36 ± 0.59	3.91 ± 0.46
	STDEV/Mean	0.363 ± 0.035	0.508 ± 0.032*
Peak Expiratory Flow (PEF) (mL/sec)	STDEV	0.575 ± 0.099	1.030 ± 0.130*
	Mean	1.85 ± 0.22	2.35 ± 0.19*
	STDEV/Mean	0.301 ± 0.025	0.426 ± 0.032*
PEF/PIF	STDEV	0.109 ± 0.016	0.133 ± 0.008
	Mean	0.529 ± 0.027	0.670 ± 0.032*
	STDEV/Mean	0.180 ± 0.020	0.200 ± 0.010
EF50 (mL/sec)	STDEV	0.027 ± 006	0.054 ± 0.008*
	Mean	0.089 ± 0.010	0.106 ± 0.010
	STDEV/Mean	0.298 ± 0.0.051	0.489 ± 0.047*
Relaxation Time (sec)	STDEV	0.019 ± 0.002	0.027 ± 0.001
	Mean	0.100 ± 0.004	0.106 ± 0.005
	STDEV/Mean	0.191 ± 0.024	0.259 ± 0.016
Expiratory Time -Relaxation Time	STDEV	0.024 ± 0.002	0.037 ± 0.002*
	Mean	0.100 ± 0.007	0.111 ± 0.005
	STDEV/Mean	0.246 ± 0.019	0.342 ± 0.021*
Inspiratory Drive (mL/sec)	STDEV	0.728 ± 0.145	1.140 ± 0.118*
	Mean	1.98 ± 0.34	2.28 ± 0.27
	STDEV/Mean	0.363 ± 0.036	0.517 ± 0.036*
Expiratory Drive (mL/sec)	STDEV	0.251 ± 0.051	0.486 ± 0.076*
	Mean	0.90 ± 0.01	1.08 ± 0.10
	STDEV/Mean	0.264 ± 0.028	0.428 ± 0.041
NEBI (%)	STDEV	18.9 ± 2.5	20.9 ± 1.0
	Mean	24.0 ± 8.0	30.3 ± 5.5
	STDEV/Mean	1.099 ± 0.160	1.031 ± 0.157
NEBI/Freq (%/(breaths/min))	STDEV	0.068 ± 0.004	0.088 ± 0.007
	Mean	0.089 ± 0.022	0.122 ± 0.020
	STDEV/Mean	0.999 ± 0.163	0.953 ± 0.131

The data are presented as mean ± SEM. *p < 0.05, HDAC6 KO *versus* WT.

### Ventilatory responses to hypoxic gas challenge and upon return to room-air

#### Frequency of breathing (Freq), tidal volume (TV), and minute ventilation (MV)

**FIGURE 1:**
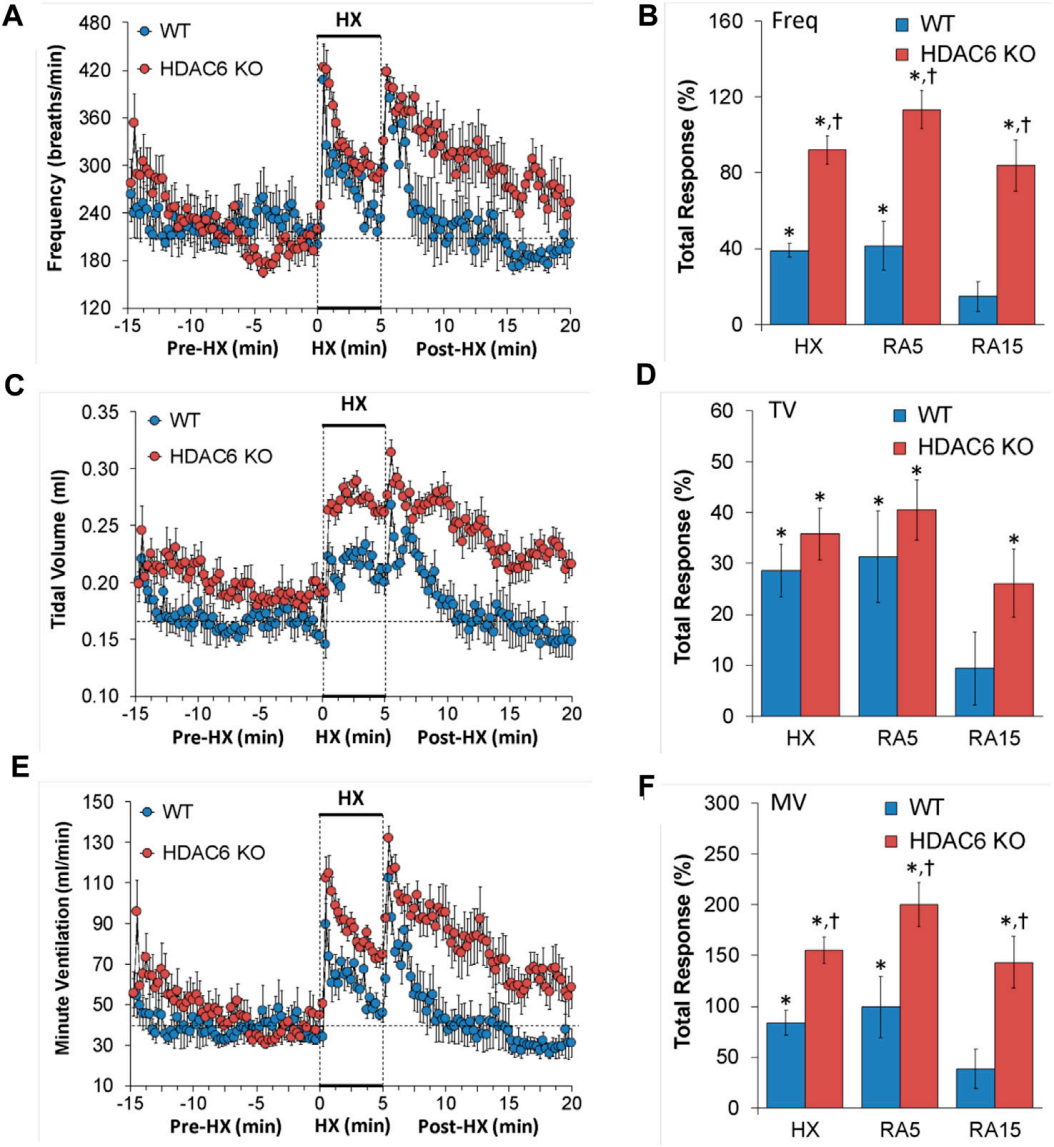
**(A,C,E)** Frequency of breathing (Freq), tidal volume (TV) and minute ventilation (MV) before (Pre-HX), during a 5 min hypoxic (HX; 10% O_2_, 90% N_2_) gas challenge, and upon return to room-air (Post-HX) in wildtype (WT) mice (*n* = 7) and HDAC6 knockout (HDAC6 KO) mice (*n* = 14). **(B,D,F)** Total responses recorded during the hypoxic (HX) gas challenge, and during the first 5 min (RA5) or during the entire 15 min (RA15) return to room-air exposure. All data are presented as mean ± SEM. *p < 0.05, significant %change from the Pre-HX values. ^†^ *p* < 0.05, significant response from HDAC6 KO *versus* WT.

Freq, TV, and MV values recorded before (Pre-HX), during a 5 min hypoxic (HX) (10% O_2_, 90% N_2_) gas challenge, and upon return to room-air (Post-HX) in WT mice and in HDAC6 KO mice are shown in the left-hand panels of Figure 1. As seen in Figure 1A, resting frequency of breathing prior to the HX gas challenge was similar in WT and HDAC6 KO mice. The HX gas challenge in WT and HDAC6 KO mice elicited typical increases in Freq associated with expected roll-off. However, the increases in Freq were higher in HDAC6 KO mice. The return to room-air elicited typical increases in Freq in the WT and HDAC6 KO mice. However, the room-air responses gradually declined over the first 5 min of the recording period in the WT mice, and corresponding Freq values were higher in HDAC6 KO mice, especially over the 5–15 min time-period when values had returned to baseline levels in WT mice. The total responses summarized in Figure 1B (see Statistics section for explanation of how the values were derived) show that the total Freq responses elicited by the HX gas challenge (HX) and upon return to room-air (RA5 and RA15) were higher in HDAC6 KO mice than in WT mice. As seen in Figure 1C, resting TV prior to HX gas challenge was consistently higher in HDAC6 KO mice than WT mice due to the slightly larger body weights of the HDAC6 KO mice (Table 1). The HX gas challenge in WT mice elicited a sustained increase in TV that did not display roll-off. The increases in TV were similarly robust in HDAC6 KO mice, however, the values were higher because of the higher resting values. Upon return to room-air, TV values returned to pre-HX levels within 5 min in WT mice but remained elevated in the HDAC6 KO mice over the 15 min recording period. The total responses summarized in Figure 1D show that the total TV responses elicited by hypoxic (HX) gas challenge were similar between HDAC6 KO and WT mice. The total TV responses upon return to room-air over the first 5 min (RA5) were similar in the HDAC6 KO and WT mice. However, the total TV responses over the entire 15 min recording period were significantly higher in HDAC6 KO mice compared to WT mice. As seen in Figure 1E, resting MV prior to the HX gas challenge was similar in WT and HDAC6 KO mice. The HX gas challenge in both the WT and HDAC6 KO mice elicited typical increases in MV that were associated with the expected roll-off. However, the increases in MV were consistently higher in HDAC6 KO mice over the 5 min gas HX gas challenge compared to WT. The return to room-air elicited a typical increase in MV in WT mice that subsided within 5 min. Moreover, the return to room-air elicited increases in MV values that were higher in HDAC6 KO mice compared to WT mice and remained elevated over the 15 min recording period. As seen in Figure 1F, the total MV responses elicited by hypoxic (HX) gas challenge and return to room-air (RA5 and RA15) were higher in the HDAC6 KO mice compared to WT mice.

#### Inspiratory time (Ti), expiratory time (Te), and Te/Ti

**FIGURE 2:**
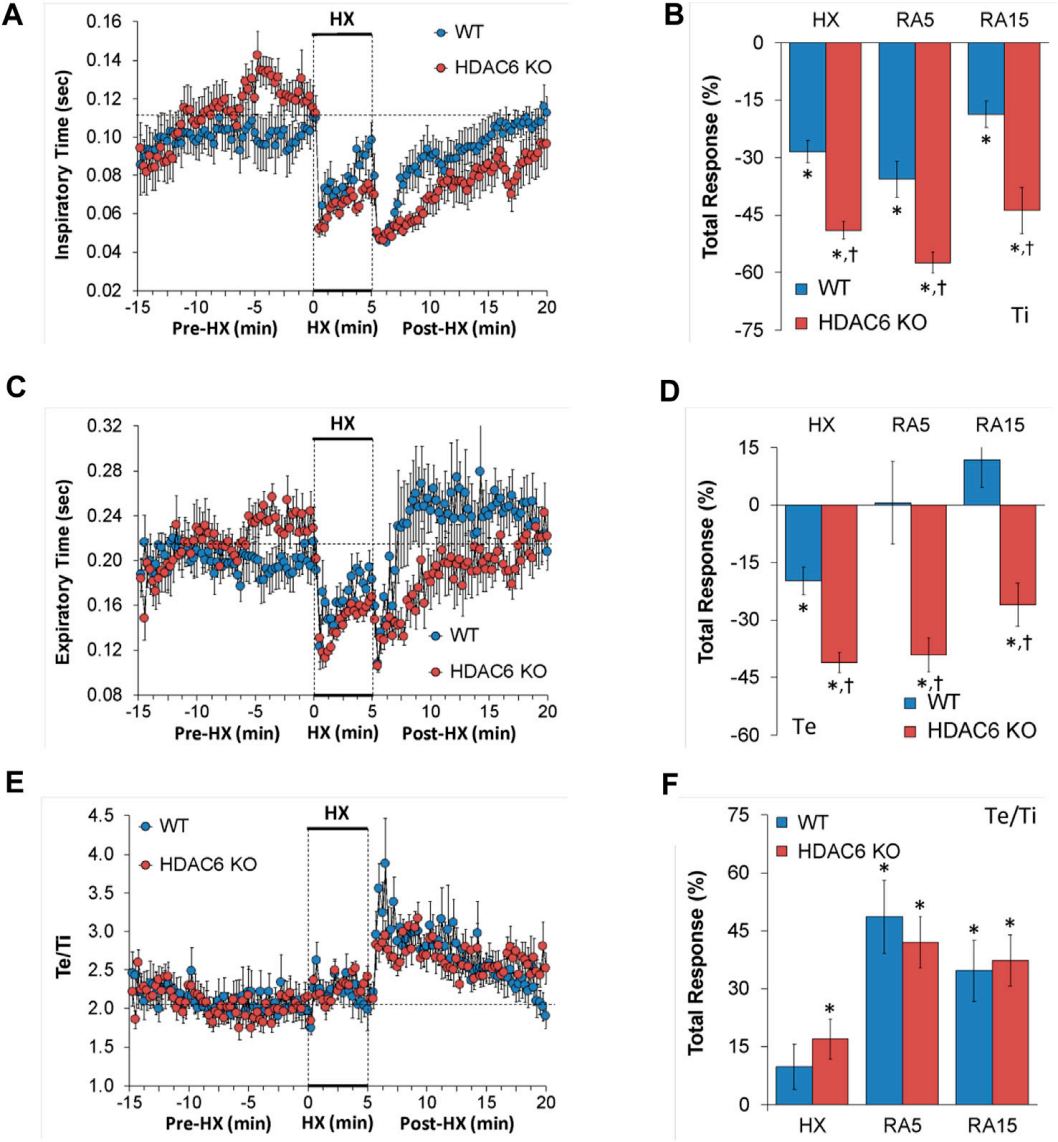
**(A,C,E)** Inspiratory time (Ti), expiratory time (Te) and Te/Ti before (Pre-HX), during a 5 min hypoxic (HX; 10% O_2_, 90% N_2_) gas challenge, and upon return to room-air (Post-HX) in wildtype (WT) mice (*n* = 7) and HDAC6 knockout (HDAC6 KO) mice (*n* = 14). **(B, D, F)** Total responses recorded during the hypoxic (HX) gas challenge, during the first 5 min (RA5) or during the entire 15 min (RA15) return to room-air exposure. All data are presented as mean ± SEM. *p < 0.05, significant %change from the Pre-HX values. ^†^ *p* < 0.05, significant response from HDAC6 KO *versus* WT.

The Ti, Te and Te/Ti values recorded before (Pre-HX), during a 5 min HX (10% O_2_, 90% N_2_) gas challenge, and upon return to room-air (Post-HX) in WT mice and in HDAC6 KO mice are shown in the left-hand panels of Figure 2. As seen in Figure 2A, resting Ti, during the 5 min period immediately prior to the HX gas challenge, was often higher in HDAC6 KO mice than WT mice. HX gas challenge in both WT and HDAC6 KO mice elicited decreases in Ti that were associated with the expected roll-off. However, the decreases in Ti were greater in HDAC6 KO mice compared to WT mice. The return to room-air elicited initial further decreases in Ti in WT mice that were followed by gradual return toward pre-HX values. Corresponding Ti values in HDAC6 KO mice showed the same pattern of changes as in WT mice but reached lower initial values that did not reach as high toward pre-HX values as the WT mice did. This phenomenon was probably because of the lower Ti values reached at the end of the hypoxic challenge. The total responses summarized in Figure 2B show that the total Ti responses elicited by hypoxic (HX) gas challenge and upon return to room-air (RA5 and RA15) were greater in HDAC6 KO mice than WT mice. As seen in Figure 2C, resting Te, during the 5 min period immediately prior to the HX gas challenge, was often higher in HDAC6 KO mice than WT mice. The HX gas challenge in both WT and HDAC6 KO mice elicited decreases in Te that were associated with expected roll-off, however, the decreases in Te were greater in HDAC6 KO mice. Return to room-air elicited an initial further decrease in Te in WT mice that was followed by a rapid return to at and above pre-HX values. Corresponding Te values in HDAC6 KO mice followed the same pattern of changes as in WT mice but stayed at lower values for longer before returning to near or at pre-HX values. The total responses summarized in Figure 2D show that total Te responses elicited by hypoxic (HX) gas challenge and upon return to room-air (RA5 and RA15) were greater in HDAC6 KO mice than in WT mice. As seen in Figure 2E, prior to HX gas challenge, resting Te/Ti was similar in HDAC6 KO and WT mice. HX gas challenge elicited minimal increases in Te/Ti in both groups compared to resting baseline values. Upon return to room-air, Te/Ti rose initially above HX values in both groups, with a spike evident in the WT mice, before gradually declining to pre-hypoxia levels. As seen in Figure 2F, the total Te/Ti responses that occurred during the hypoxic challenge (HX) and upon return to room-air (RA5 and RA15) were similar in HDAC6 KO and WT mice.

#### End inspiratory pause (EIP) and end expiratory pause (EEP)

**FIGURE 3:**
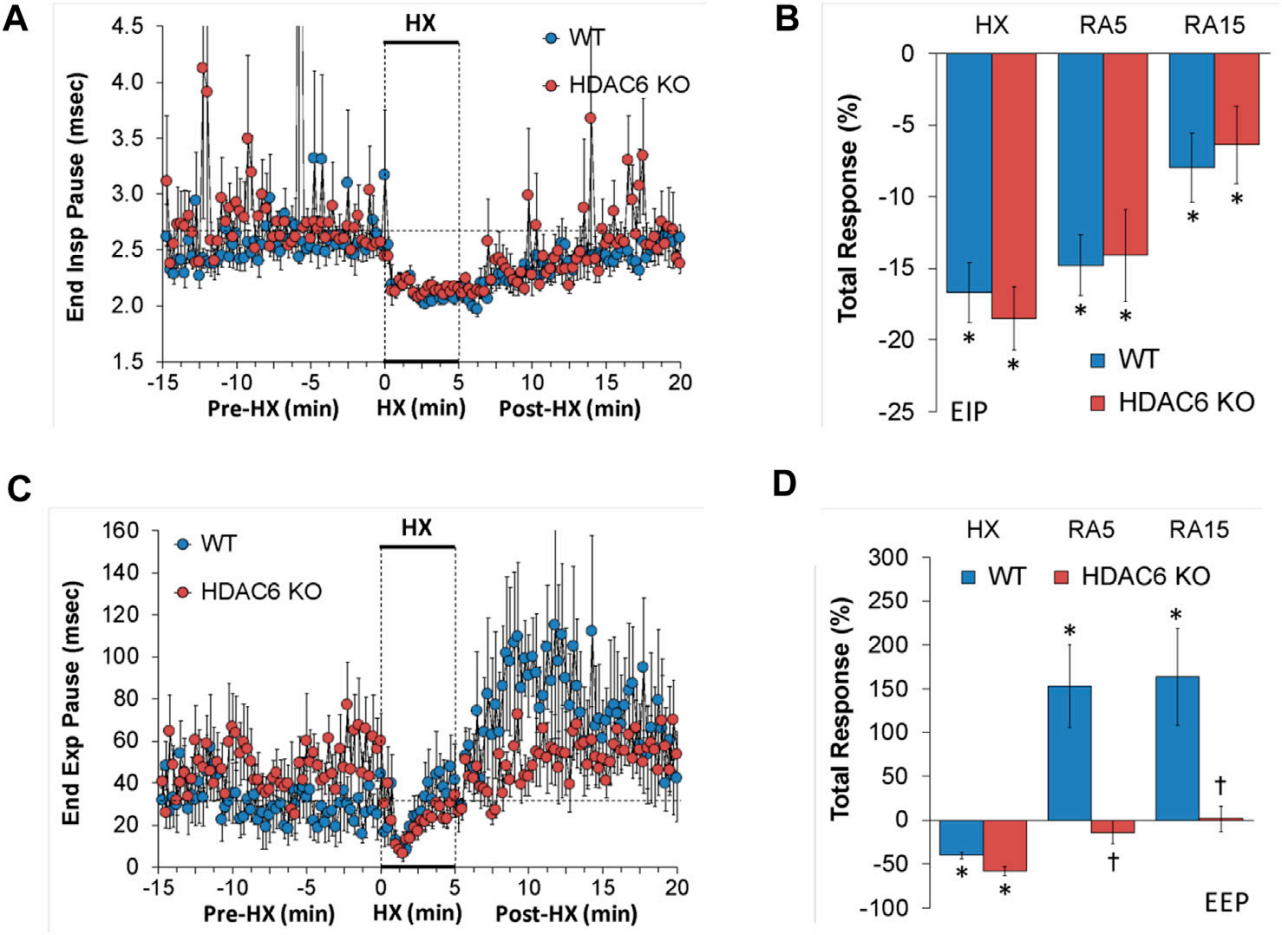
**(A,C)** End inspiratory pause (End Insp Pause, EIP) and end expiratory pause (End Exp Pause, EEP) before (Pre-HX), during a 5 min hypoxic (HX; 10% O_2_, 90% N_2_) gas challenge, and upon return to room-air (Post-HX) in wildtype (WT) mice (*n* = 7) and HDAC6 knockout (HDAC6 KO) mice (*n* = 14). **(B,D)** Total responses recorded during the hypoxic (HX) gas challenge, during the first 5 min (RA5) or during the entire 15 min (RA15) return to room-air exposure. All data are presented as mean ± SEM. *p < 0.05, significant %change from the Pre-HX values. ^†^ *p* < 0.05, significant response from HDAC6 KO *versus* WT.

EIP and EEP values recorded before (Pre-HX), during a 5 min HX (10% O_2_, 90% N_2_) gas challenge, and upon return to room-air (Post-HX) in WT mice and HDAC6 KO mice are shown in the left-hand panels of Figure 3. As seen in Figure 3A, resting EIP and EEP values prior HX gas challenge were similar in HDAC6 KO mice and WT mice. The HX gas challenge elicited similar sustained decreases in EIP in WT and HDAC6 KO mice. Upon return to room-air, EIP values gradually returned to pre-HX levels in both groups. As seen in Figure 3B, the total EIP responses elicited by hypoxic (HX) gas challenge and upon return to room-air (RA5 and RA15) were similar in HDAC6 KO and WT mice. As seen in Figure 3C, resting EEP values were variable and often higher in HDAC6 KO mice. The HX gas challenge elicited an initial decrease in EEP in both groups that recovered toward baseline at the end of the challenge. Upon return to room-air, EEP rose above baseline levels in WT mice, but stayed near baseline values in the HDAC6 KO mice. As seen in Figure 3D, the total decreases in EEP elicited by the HX gas challenge were similar in WT and HDAC6 KO mice. In contrast, the total increases in EEP observed upon return to room-air in WT mice (RA5 and RA15) were not seen in the HDAC6 KO mice.

#### Peak inspiratory flow (PIF), peak expiratory flow (PEF), and PEF/PIF

**FIGURE 4:**
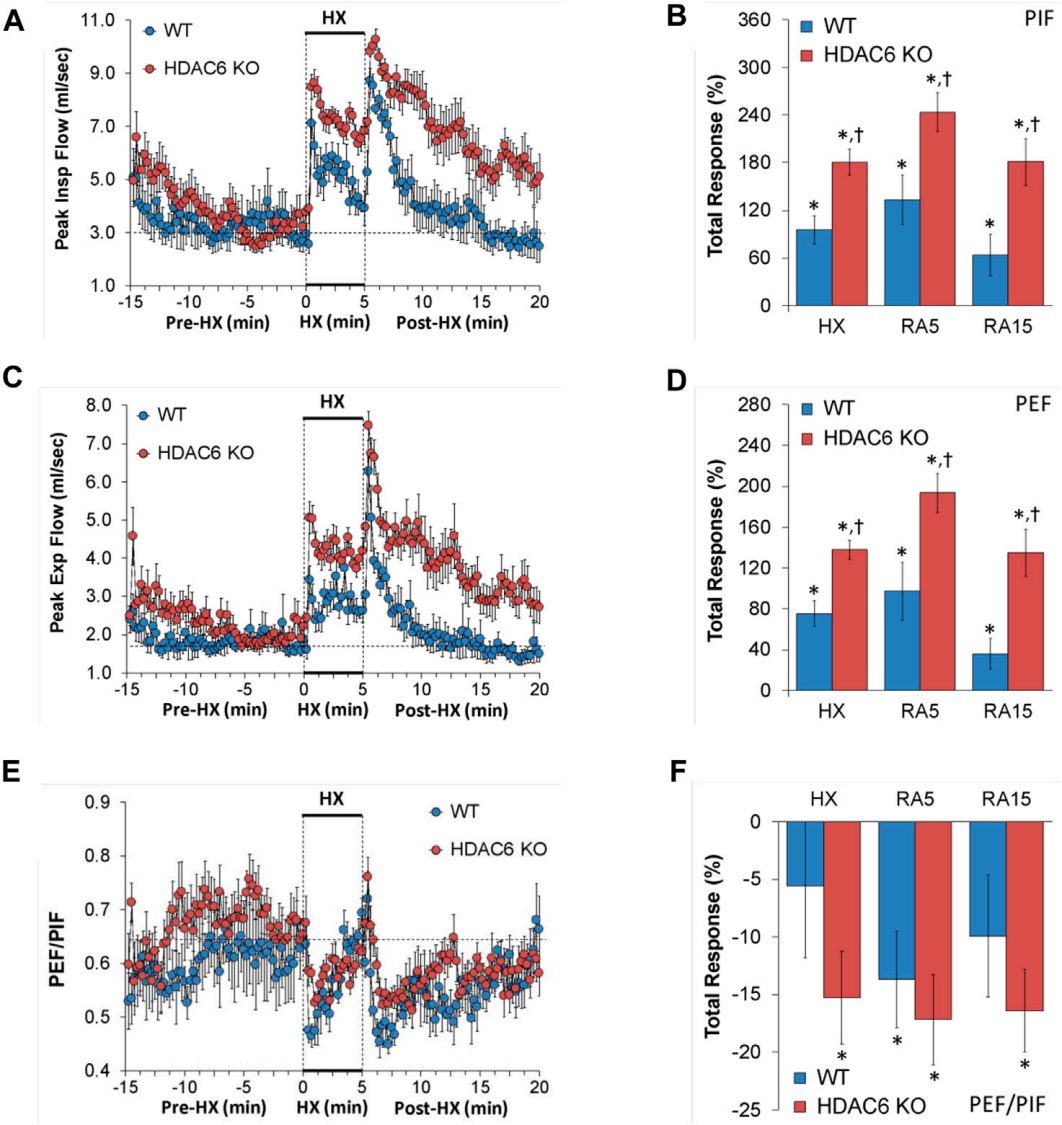
**(A,C,E)** Peak inspiratory flow (Peak Insp Flow, PIF), peak expiratory flow (Peak Exp Flow, PEF), and PEF/PIF before (Pre-HX), during a 5 min hypoxic (HX; 10% O_2_, 90% N_2_) gas challenge, and upon return to room-air (Post-HX) in wildtype (WT) mice (*n* = 7) and HDAC6 knockout (HDAC6 KO) mice (*n* = 14). **(B,D,F)** Total responses recorded during the hypoxic (HX) gas challenge, during the first 5 min (RA5) or during the entire 15 min (RA15) return to room-air exposure. All data are presented as mean ± SEM. *p < 0.05, significant %change from the Pre-HX values. ^†^ *p* < 0.05, significant response from HDAC6 KO *versus* WT.

PIF and PEF values recorded before (Pre-HX), during a 5 min HX (10% O_2_, 90% N_2_) gas challenge, and upon return to room-air (Post-HX) in WT mice and HDAC6 KO mice are shown in the left-hand panels of Figure 4. As seen in Figures 4A, C, resting PIF and PEF values were similar between the HDAC6 KO mice and WT mice. The HX gas challenge elicited sustained increases in PIF and PEF in both WT mice and HDAC6 KO mice. However, the PIF and PEF responses during the HX gas challenge were markedly augmented in HDAC6 KO mice. Upon return to room-air, PIF and PEF spiked upward initially before gradually returning to resting baseline levels in WT mice. The HDAC6 KO mice followed a similar PIF and PEF response pattern upon return to room-air, however the PIF and PEF values remained higher, and did not reach resting baseline levels as the WT mice did after 15 min. As seen in Figures 4B, D, total PIF and PEF responses elicited by hypoxic (HX) gas challenge and upon return to room-air (RA5 and RA15) were greater in the HDAC6 KO mice than in the WT mice.

#### EF_50_, relaxation time, and expiratory delay (Te-RT)

**FIGURE 5:**
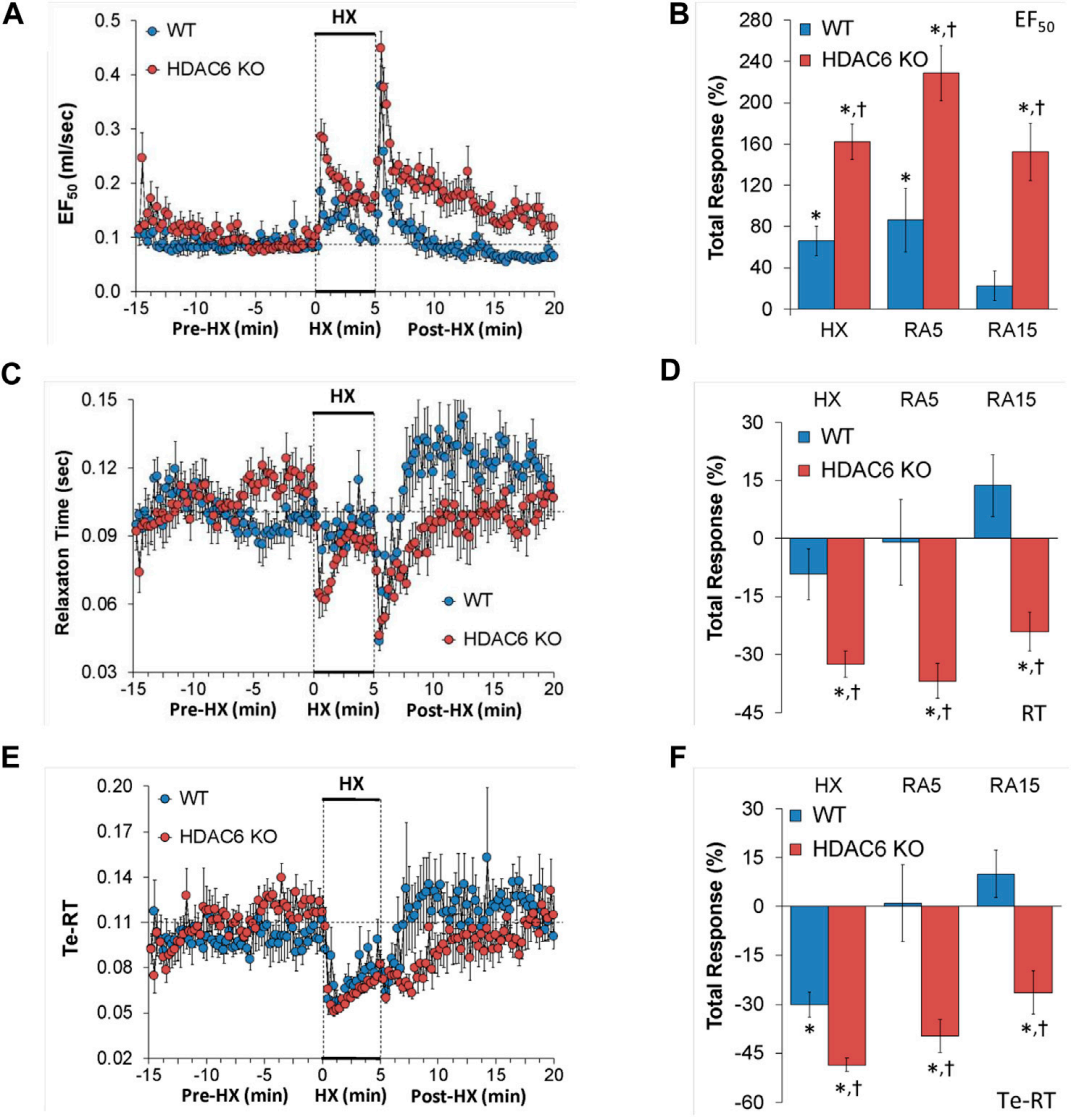
**(A,C,E)** Expiratory flow at 50% tidal volume (EF_50_), relaxation time (RT), and expiratory delay (Te-RT) before (Pre-HX), during a 5 min hypoxic (HX; 10% O_2_, 90% N_2_) gas challenge, and upon return to room-air (Post-HX) in wildtype (WT) mice (*n* = 7) and HDAC6 knockout (HDAC6 KO) mice (*n* = 14). **(B,D,F)** Total responses recorded during the hypoxic (HX) gas challenge, during the first 5 min (RA5) or during the entire 15 min (RA15) return to room-air exposure. All data are presented as mean ± SEM. *p < 0.05, significant %change from the Pre-HX values. ^†^ *p* < 0.05, significant response from HDAC6 KO *versus* WT.

EF_50_, relaxation time, and expiratory delay (Te-RT) values recorded before (Pre-HX), during a 5 min HX (10% O_2_, 90% N_2_) gas challenge, and upon return to room-air (Post-HX) in WT mice and HDAC6 KO mice are shown in the left-hand panels of Figure 5. As seen in Figure 5A, resting EF_50_ values were similar in HDAC6 KO mice and WT mice. The HX gas challenge elicited greater increases in EF_50_ in HDAC6 KO mice than in WT mice. The responses that occurred upon return to room-air were also greater in HDAC6 KO mice. As seen in Figure 5B, the total increases EF_50_ elicited by HX gas challenge and upon return to room-air (RA5 and RA15) were greater in HDAC6 KO than in WT mice. As seen in Figure 5C, resting relaxation time values were similar in HDAC6 KO mice and WT mice. The HX gas challenge elicited minimal changes in relaxation time in WT mice, but substantial initial falls in the HDAC6 KO mice group that recovered within 3 min of the hypoxia exposure. Upon initial return to room-air, relaxation time dropped substantially in both WT and HDAC6 KO mice. After initial room-air exposure, relaxation time then rose above baseline values in WT mice but returned to baseline values in HDAC6 KO mice. As seen in Figure 5D, total decreases in relaxation elicited by HX and upon return to room-air (RA5 and RA15) were greater in HDAC6 KO than in WT mice. As seen in Figure 5E, resting expiratory delay (Te-RT) values were higher in HDAC6 KO mice compared to WT mice for approximately 5 min before the HX gas challenge. The HX gas challenge elicited slightly greater decreases in expiratory delay in HDAC6 KO mice compared to WT mice. Expiratory delay returned rapidly to at or slightly above baseline levels in WT mice, whereas these values remained below baseline values in HDAC6 KO mice for 5–6 min before returning to near or at baseline levels. As seen in Figure 5F, the total decreases in expiratory delay elicited during HX gas challenge were greater in HDAC6 KO mice compared to WT mice. Decreases in expiratory delay upon return to room-air (RA5 and RA15) occurred in HDAC6 KO mice only (Figure 5F).

#### Inspiratory drive (TV/Ti) and expiratory drive (TV/Te)

**FIGURE 6:**
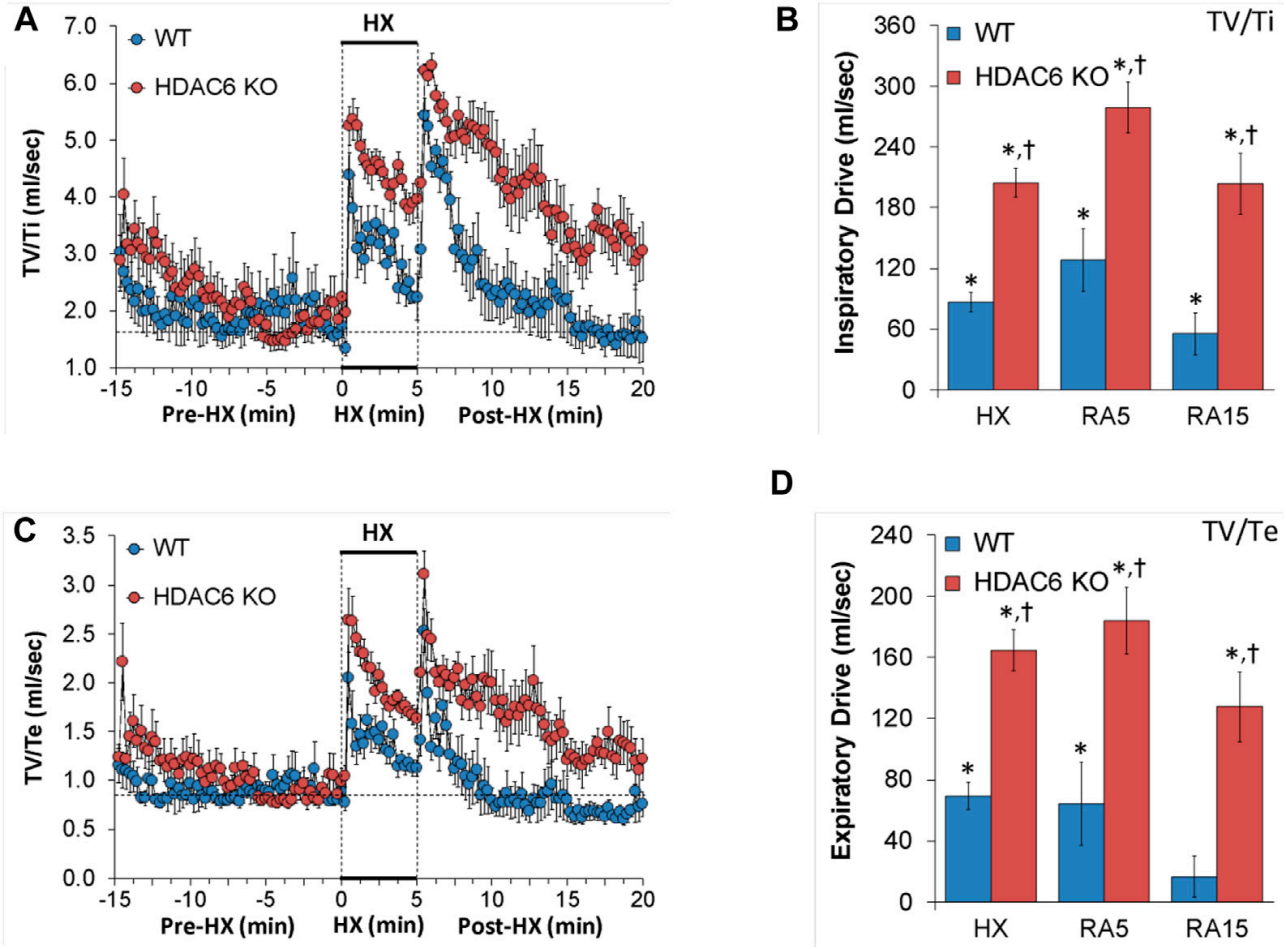
**(A,C)** Inspiratory drive (tidal volume/inspiratory time, TV/Ti) and expiratory time (tidal volume/expiratory time, TV/Te) before (Pre-HX), during a 5 min hypoxic (HX; 10% O_2_, 90% N_2_) gas challenge, and upon return to room-air (Post-HX) in wildtype (WT) mice (*n* = 7) and HDAC6 knockout (HDAC6 KO) mice (*n* = 14). **(B,D)** Total responses recorded during the hypoxic (HX) gas challenge, during the first 5 min (RA5) or during the entire 15 min (RA15) return to room-air exposure. All data are presented as mean ± SEM. *p < 0.05, significant %change from the Pre-HX values. ^†^ *p* < 0.05, significant response from HDAC6 KO *versus* WT.

Inspiratory drive and expiratory drive values recorded before (Pre-HX), during a 5 min HX (10% O_2_, 90% N_2_) gas challenge, and upon return to room-air (Post-HX) in WT mice and HDAC6 KO mice are shown in the left-hand panels of Figure 6. As seen in Figures 6A, C, resting inspiratory and expiratory drives prior to HX gas challenge were similar in HDAC6 KO mice and WT mice. The HX gas challenge elicited sustained increases in inspiratory drive and expiratory drive in WT mice. Additionally, the HX gas challenge elicited sustained increases in inspiratory and expiratory drives in HDAC6 KO mice, however these responses were augmented. Upon return to room-air, inspiratory drive and expiratory drive values spiked upward in WT mice before gradually returning to at or near baseline levels. Moreover, upon return to room-air, inspiratory drive and expiratory drive values spiked upward in HDAC6 KO mice, however, although these responses gradually declined, they did not return to at or near baseline levels. As seen in Figures 6B, D, the responses elicited during the HX gas challenge and upon return to room-air (RA5 and RA15) were greater in HDAC6 KO mice compared to WT mice.

#### Non-eupneic breathing index (NEBI) and NEBI/Freq

**FIGURE 7:**
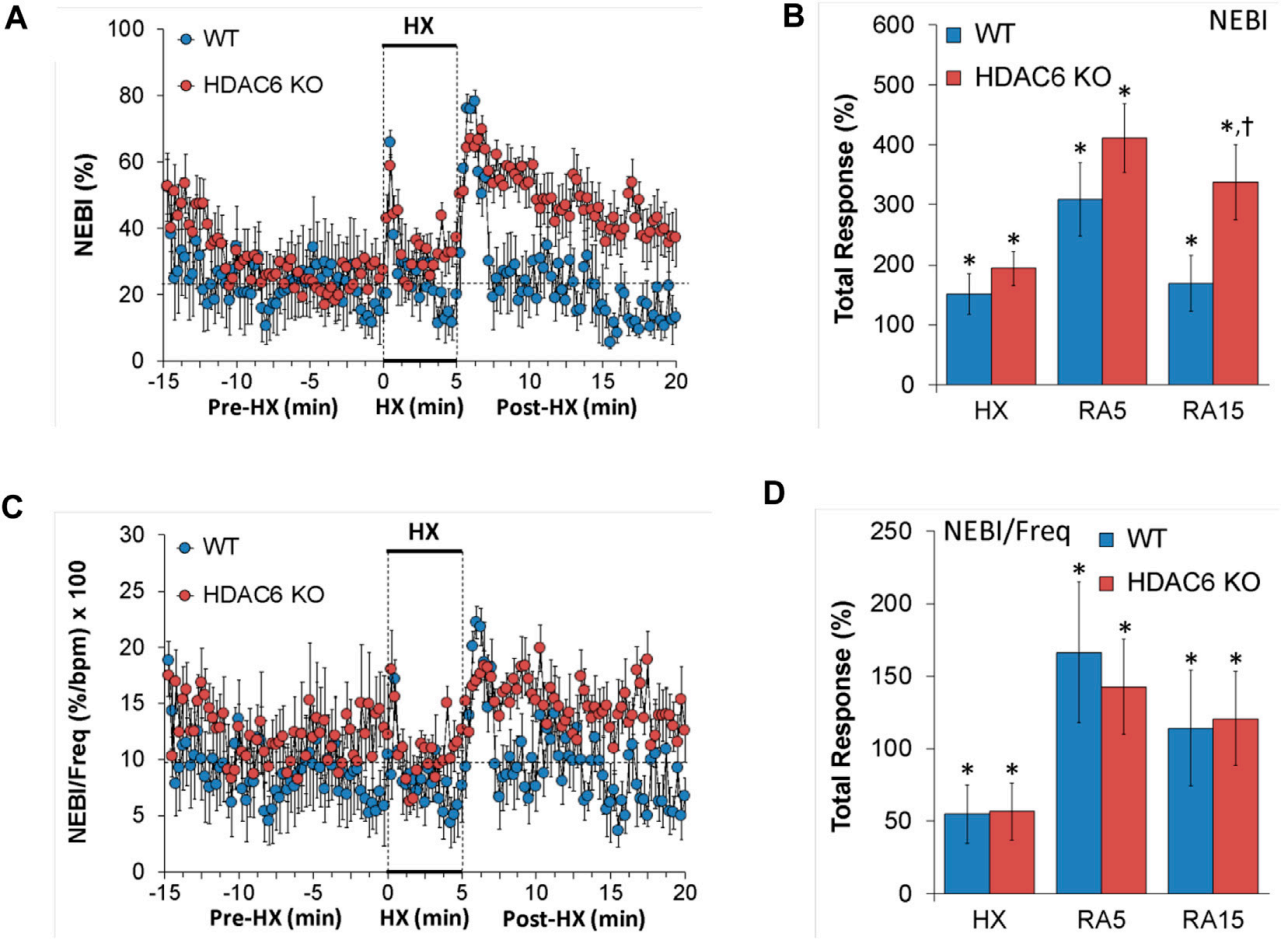
**(A,C)** Non-eupneic breathing index (NEBI) and NEBI/frequency of breathing (NEBI/Freq) before (Pre-HX), during a 5 min hypoxic (HX; 10% O_2_, 90% N_2_) gas challenge and upon return to room-air (Post-HX) in wildtype (WT) mice (*n* = 7) and HDAC6 knockout (HDAC6 KO) mice (*n* = 14). **(B,D)** Total responses recorded during the hypoxic (HX) gas challenge, during the first 5 min (RA5) or during the entire 15 min (RA15) return to room-air exposure. All data are presented as mean ± SEM. *p < 0.05, significant %change from the Pre-HX values. ^†^ *p* < 0.05, significant response from HDAC6 KO *versus* WT.

NEBI and NEBI/Freq values recorded before (Pre-HX), during a 5 min HX (10% O_2_, 90% N_2_) gas challenge, and upon return to room-air (Post-HX) in WT mice and HDAC6 KO mice are shown in the left-hand panels of Figure 7. As seen in Figure 7A, resting NEBI values prior to the HX gas challenge were similar in HDAC6 KO mice and WT mice. The HX gas challenge elicited similar increases in NEBI in WT mice and HDAC6 KO mice. The return to room-air caused remarkable increases in NEBI in both groups, with NEBI subsiding more rapidly to at or near baseline levels in WT mice, but the NEBI values remaining considerable higher in HDAC6 KO mice. As seen in Figure 7B, the increases in NEBI elicited during the HX gas challenge, and during the first 5 min upon return to room-air (RA5) were similar in the WT and HDAC6 KO mice, whereas the overall increase in NEBI during the entire 15 min room-air exposure (RA15) was greater in the HDAC6 KO mice. As seen in Figures 7C, D, normalizing the changes in NEBI for the changes in frequency of breathing (NEBI/Freq) resulted in changes during hypoxic (HX) gas challenge and upon return to room-air (RA5 and RA15) that were similar in both groups of mice.

#### Body weight considerations

The heavier body weights of HDAC6 KO mice may influence findings related to the effects of HX gas challenge and return to room-air on flow parameters; TV, MV, peak PIF, PEF. and EF_50_. Table 3 summarizes the total arithmetic changes in ventilatory parameters during HX gas challenge with flow parameters also shown corrected for body weight. Total arithmetic changes in Freq, Ti, Te, Te/Ti, EIP, EEP, relaxation time, Te-relaxation time, NEBI, and NEBI/Freq corrected for body weight, provide the same conclusions given by the %change data in Figures 1–7, suggesting that HX-mediated changes in these parameters were greater in HDAC6 KO mice compared to WT mice. Second, the delta changes in flow variables corrected for body weight (delta/body weight)—TV, MV, PIF, PEF, EF_50_, and inspiratory and expiratory drives—were also consistent with %change data provided in Figures 1–7 in that, except for TV, the HX-mediated changes in these parameters were greater in HDAC6 KO mice than in WT mice.

**TABLE 3:** Total arithmetic responses during hypoxic gas challenge.

Parameters	Response	WT mice	HDAC6 KO mice
Flow-independent			
Frequency (breaths/min)	Delta response	+1,570 ± 157*	+3,076 ± 212*^,†^
Inspiratory Time (sec)	Delta response	−0.63 ± 0.07*	−1.33 ± 0.09*^,†^
Expiratory Time (sec)	Delta response	−0.85 ± 0.16*	−2.12 ± 0.19*^,†^
Expiratory Time/Inspiratory Time	Delta response	+3.72 ± 2.27	+5.66 ± 1.82*
End Inspiratory Pause (msec)	Delta response	−8.84 ± 1.44*	−10.28 ± 1.41*
End Expiratory Pause (msec)	Delta response	−227 ± 54*	−781 ± 153*^,†^
Relaxation Time (sec)	Delta response	−0.22 ± 0.13	−0.82 ± 0.11*^,†^
Expiratory Time–Relaxation Time	Delta response	−0.63 ± 0.07*	−1.26 ± 0.08*^,†^
NEBI (%)	Delta response	+292 ± 55*	+420 ± 40*^,†^
NEBI/Freq [%/(breaths/min)]	Delta response	+0.46 ± 0.17*	+0.40 ± 0.24*
Flow-dependent			
Tidal Volume (mL)	Delta response	+0.91 ± 0.14*	+1.34 ± 0.15*
	Delta/Body Weight	+0.033 ± 0.005*	+0.045 ± 0.006
Minute Ventilation (mL/min)	Delta response	+531 ± 74*	+1,024 ± 54*^,†^
	Delta/Body Weight	+19.3 ± 2.5*	+33.6 ± 2.2*^,†^
PIF (mL/sec)	Delta response	+46.1 ± 6.6*	+88.5 ± 4.3*^,†^
	Delta/Body Weight	+1.67 ± 0.22*	+2.90 ± 0.17*^,†^
PEF (mL/sec)	Delta response	+23.6 ± 3.4*	+47.7 ± 2.7*^,†^
	Delta/Body Weight	+0.863 ± 0.12*	+1.55 ± 0.09*^,†^
PEF/PIF	Delta response	−0.93 ± 0.79	−2.39 ± 0.51
	Delta/Body Weight	−0.032 ± 0.029	−0.081 ± 0.016
EF_50_ (mL/sec)	Delta response	+0.98 ± 0.18*	+2.34 ± 0.16*^,†^
	Delta/Body Weight	0.035 ± 0.006*	+0.076 ± 0.006*^,†^
Inspiratory Drive (mL/sec)	Delta response	+26.9 ± 2.4*	+57.5 ± 3.0*^,†^
	Delta/Body Weight	+0.99 ± 0.08*	+1.88 ± 0.11*^,†^
Expiratory Drive (mL/sec)	Delta response	+11.1 ± 1.6*	24.3 ± 1.7*^,†^
	Delta/Body Weight	+0.40 ± 0.05*	+0.80 ± 0.06*^,†^

WT, wildtype; HDAC6 KO, histone deacetylase 6 knockout mice; NEBI, non-eupneic breathing index; Freq, frequency of breathing; PIF, peak inspiratory flow; PEF, peak expiratory flow; EF_50_, airflow at 50% expired tidal volume.

The data are presented as mean ± SEM. *p < 0.05, significant %change from the Pre-HX, values. †*p* < 0.05, significant response from HDAC6 KO *versus* WT.

## Discussion

The C57BL/6 mouse strain is a common inbred strain that is widely used in pulmonary studies (Soliz et al., 2008; Soliz et al., 2009) and to produce mice lacking genes for numerous functional proteins involved in respiratory mechanics (Kline et al., 2002; Liu et al., 2004; Duling et al., 2006; Palmer et al., 2013b). Because it is a popular inbred strain, the C57BL/6 mouse is displayed as having “normal” physiology and indeed they display many “normal” traits (Tankersley et al., 2002a; Tankersley et al., 2002b; Tankersley et al., 2002c; Campen et al., 2004; Campen et al., 2005; Palmer et al., 2013b; Palmer et al., 2015; Tewari et al., 2013; Gaston et al., 2014; Getsy et al., 2014). For example, systemic and pulmonary arterial blood pressures and cardiovascular responses of C57BL/6 mice upon challenges with hypoxic, hypercapnic, and hypoxic-hypercapnic gases are representative of other healthy mouse and rat strains (Campen et al., 2004; Campen et al., 2005; Tewari et al., 2013). As such, C57BL/6 mice have been used extensively to study the effects of hypoxic, hypercapnic, and hypoxic-hypercapnic gas challenges on ventilatory function (Palmer et al., 2013a; Palmer et al., 2013b; Gaston et al., 2014; Getsy et al., 2014; Palmer et al., 2015), and disordered breathing during both wakefulness and sleep (Han et al., 2001; Han et al., 2002; Tagaito et al., 2001; Schneider et al., 2003; Yamauchi et al., 2007; Yamauchi et al., 2008a; Yamauchi et al., 2008b; Yamauchi et al., 2010; Moore et al., 2012; Moore et al., 2014). Despite being younger, HDAC6 KO mice were slightly heavier than their WT (C57BL/6) littermate controls. Whether this means that deletion of HDAC6 affects body metabolism or other factors regulating general health/body weight in C56BL/6 mice are yet to be established. The loss of HDAC6 could directly and/or indirectly impact ventilatory parameters in C57BL/6 mice by numerous mechanisms. For example, HDAC6 exists in smooth muscle and vascular endothelium of pulmonary arteries, and inhibition of HDAC6 improves the functions of both cell types (Joshi et al., 2015; Boucherat et al., 2017).

### Resting ventilatory parameters

A key finding of this study was that resting baseline (Pre-HX gas challenge) Freq values were lower in the HDAC6 KO mice than in WT mice. The reduced baseline Freq values in HDAC6 KO mice was accompanied by longer resting Ti and Te values, suggesting that the possible presence of HDAC6 in key brainstem sites controlling respiratory frequency, such as the NTS, has a vital role in setting resting Ti and Te. Similarly, the findings that EEP and expiratory delay (Te-RT) were greater in HDAC6 KO mice, suggests that HDAC6 is important for regulation of expiratory dynamics. Finally, the findings that the majority of the resting ventilatory parameters were similar in HDAC6 KO and WT mice (e.g., TV, PIF, and PEF) does not negate a role for HDAC6 in control of these parameters, but rather that C57BL/6 mice can compensate for the loss of HDAC6.

### Ventilatory responses to hypoxic (HX) gas challenge

The HX gas challenge elicited greater increases in Freq, but not TV, and MV, in HDAC6 KO mice compared to WT mice. These findings suggest that stabilizing microtubules has a very important positive effect on respiratory timing, but perhaps not ventilatory mechanics. The carotid body and chemoafferents in the carotid sinus nerve play an essential role in detecting and transmitting hypoxic signals to the commissural nuclei tractus solitarii in the brainstem (Lahiri et al., 2006; Prabhakar and Peers, 2014; López-Barneo et al., 2016; Baby et al., 2018). Previously, we have reported that hypoxic ventilatory responses are markedly reduced in male C57BL/6 (WT) mice with bilateral carotid sinus nerve transection (Gaston et al., 2014). Although currently lacking, any evidence that HDAC6 exists in the carotid bodies and key brain structures, such as the nucleus tractus solitarii, would support our evidence that HDAC6 is vital to hypoxic ventilatory signaling. The hypoxia-induced increases in Freq were, as expected, associated with temporally consistent decreases in inspiratory and expiratory times in WT and HDAC6 KO mice. The decreases in Ti and Te were greater in HDAC6 KO mice than in WT mice, consistent with the more pronounced increases in Freq in HDAC6 KO mice. The decrease in Ti was greater than the decrease in Te in both the WT and HDAC6 KO mice, such that there was a slight increase in expiratory quotient (Te/Ti) in both groups of mice, with the increase in this ratio being larger in HDAC6 KO mice. The combinations of increased TV coupled to decreases in Ti and Te resulted in marked increases in inspiratory drive (TV/Ti) and expiratory drive (TV/Te) in both groups, but which were substantially larger in the HDAC6 KO mice. Again, although data is not available regarding the presence of HDAC6 in brain sites that participate in hypoxic signaling, it is known that HDAC6 exists throughout the brain, and is particularly associated with serotonergic neurons, such as the dorsal and median raphe nuclei (Espallergues et al., 2012; Fukada et al., 2012; Tang et al., 2017) that are known to have important roles in the control of ventilatory processes (Hilaire et al., 2010; Pilowsky, 2014; Teran et al., 2014). Nonetheless, previous research suggests that serotonergic neurons play a key role in the expression of the ventilatory responses to hypercapnic gas challenges, but not a major role in the expression of ventilatory responses to hypoxic gas challenges (Richerson et al., 2001; Taylor et al., 2005; Hodges et al., 2008; Hodges and Richerson, 2008; Li and Nattie, 2008; Nattie, 2008; Nucci et al., 2008).

As expected, EIP and EEP decreased during exposure to hypoxic gas challenge in both WT and HDAC6 KO mice, and these decreases in EIP and EEP were similar in WT and HDAC6 KO mice. In addition, although relaxation time and expiratory delay (Te - relaxation time) shortened remarkably during the hypoxic gas challenge in both groups, the decreases were substantially greater in HDAC6 KO mice compared to WT mice. Moreover, the increases in PIF, PEF, and EF_50_ during the hypoxic gas challenge were augmented in HDAC6 KO mice compared to WT mice. Again, although it is not known if HDAC6 exists in the diaphragm and/or chest wall, histone deacetylases do exist in skeletal muscle (Poddar et al., 2016; Luo et al., 2019; Song et al., 2019), and as such, reduced expression and/or pharmacological blockade of HDAC6 may increase the force of contraction generated by ventilatory muscles, thereby enhancing PIF, PEF, and EF_50_ responses during hypoxic gas challenge. Finally, the hypoxic gas challenge caused a substantially greater increase in NEBI (e.g., disordered breathing, apneas, type 1 and 2 sighs) of HDAC6 KO mice compared to WT mice, although when corrected for Freq values, NEBI/Freq was similar in both groups. Previously, we have argued that NEBI may reach higher values with higher levels of Freq (Getsy et al., 2014; Getsy et al., 2023a; Getsy et al., 2023b), this may not always be the case, and so it is plausible that the lack of HDAC6 destabilizes breathing patterns during hypoxic gas challenge.

### Ventilatory responses upon return to room-air

Return to room-air in mice having undergone exposure to hypoxic gas challenge often results in abrupt dramatic increases in Freq and TV, and therefore MV (Powell et al., 1998; Palmer et al., 2013a; Palmer et al., 2013b; Palmer et al., 2015; Gaston et al., 2014; Getsy et al., 2014; Getsy et al., 2023a; Getsy et al., 2023b) that can result in unstable breathing (Powell et al., 1998; Strohl, 2003; Yamauchi et al., 2008a; Yamauchi et al., 2008b). Mechanisms responsible for post-HX alterations in breathing have received considerable investigation, and at present, evidence favors disturbances in central signaling (Wilkinson et al., 1997; Strohl, 2003), including the pons (Coles and Dick, 1996; Dick and Coles, 2000), rather than processes within the carotid bodies (Vizek et al., 1987; Brown et al., 1993). The present study found that C57BL/6 WT mice displayed the expected abrupt increase Freq, TV, and MV upon return to room-air, which recovered to baseline values within 5 min. The increases in Freq and MV, but not TV, upon return to room-air were greater in HDAC6 KO mice than WT mice over the first 5 min of return to room-air and took substantially longer to recover to baseline values. These results suggest that the presence of HDAC6, within peripheral and central neural structures, plays a vital role in the ventilatory adaptations that occur upon the return to room-air. As would be expected, the decreases in Ti and Te were greater in HDAC6 KO mice than the WT mice over the first 5 min following return to room-air. A careful review of the data shows that Te returned to pre-HX (baseline) values relatively abruptly in WT mice, whereas it remained decreased for approximately 5–10 min in HDAC6 KO mice before returning to near baseline values. This evidence is strongly supported by the abrupt and sustained increases in EEP that occurred upon return to room-air in WT mice, that were virtually absent in HDAC6 KO mice. Contrarily, our data shows a gradual return of EIP to baseline levels upon return to room-air in both WT and HDAC6 KO mice, suggesting that HDAC6 has a major role in brain neural circuitry regulating expiratory timing over inspiratory timing. The increases in PIF, PEF, and EF_50_ upon return to room-air were greater over the first 5 min in HDAC6 KO mice than in WT mice and remained greater for longer periods of time during room-air recovery. Again, enhanced activity of skeletal muscle in the chest wall and diaphragm may be directly responsible for the enhanced responses in HDAC6 KO mice, although augmented central output to these muscles cannot be discounted. The findings that the decreases in relaxation time and expiratory delay (Te-RT) were remarkably greater in the HDAC6 KO mice, also points to an important role for HDAC6 in expiratory control processes. Taking the changes in TV, Ti and Te into account, it was evident that the increases in inspiratory drive (TV/Ti) and expiratory drive (TV/Te) upon return to room-air were greater in HDAC6 KO mice. Taken together, the data reinforce the overall impression that HDAC6 has a major role in regulating inspiratory and expiratory timing in C57BL/6 mice during hypoxia exposure. The finding that the increase in NEBI upon return to room-air was greater in the HDAC6 KO mice tentatively suggests that HDAC6 plays a vital role in ventilatory stability during return to room-air following hypoxic gas challenge, and that the loss of HDAC6 may contribute to ventilatory instability (i.e., increased expression of abnormal breaths and apneas). The finding that post-hypoxia (i.e., post-apnea) breathing in humans is associated with severe glottal closures (Dempsey et al., 2010; Strohl et al., 2012), raises the possibility that decreased expression of HDAC6 may contribute to obstruction of the upper airway in patients with obstructive apneas, and perhaps the expression of central apneas.

## Study limitations

The results of the present study in male C57BL/6 mice raises the question of whether female HDAC6 KO mice will display many of the ventilatory features displayed by male mice. Therefore, an important limitation of this study was that only male C57BL/6 and HDAC6 KO mice were used and not female mice to evaluate the hypoxic ventilatory responses and those that occur upon return to room-air. This is important because previous studies have uncovered important sex differences in the ventilatory responses of mice during and following HX gas challenges (Palmer et al., 2013a; Palmer et al., 2013b). At the present time, it is unknown why HDAC6 KO mice display such marked differences in their responses to the HX gas challenge. Presumably, HDAC6 plays a critical role in signaling events within central and peripheral neural pathways involved in expressing ventilatory responses to hypoxic challenge. The loss of HDAC6 in signaling pathways involved in respiration may be responsible for altered responsiveness of these mice and morphological/functional changes in key ventilatory structures, such as the carotid body (Baby et al., 2004; Chai et al., 2011) and upper airway (Strohl et al., 2012). Additionally, future studies need to evaluate the ventilatory responses in male and female mice to a hypercapnic gas challenge to determine whether the loss of HDAC6 in key brainstem structures responsible for responding to hypercapnia, such as the retrotrapezoid nucleus-parafacial complex (Guyenet and Mulkey, 2010; Guyenet and Bayliss, 2022), qualitatively and/or quantitatively alters the expression of the responses and if the changes are sex dependent. Currently, the mechanism(s) by which HDAC6 KO mice have altered ventilatory parameters during baseline recording, including Freq (lower than in WT mice), Ti (higher than in WT mice), Te (higher than in WT mice), EEP (longer than in WT mice), and expiratory delay (longer than in WT mice), are not well-understood. Moreover, it is unknown why the ventilatory responses during and following HX gas challenge were so different in HDAC6 KO compared to WT mice. It would seem apparent that either the loss of HDAC6 itself or changes in the expression of other key signaling molecules resulting from the loss of HDAC6 are involved. Additionally, changes in the expression of HDAC6 or down-stream elements vital to the hypoxic ventilatory response in the carotid body and/or commissural nucleus tractus solitarius would likely be important areas of focus. Previous research has shown a strong interplay between HDAC6 and HIF-1α (Kong et al., 2006; Qian et al., 2006; Su et al., 2016; Leng et al., 2018), and both HDAC6 (Chen et al., 2008; Höhn et al., 2013; Seidel et al., 2015; Lkhagva et al., 2018) and HIF-1α (Kim et al., 2006; Choudhry and Harris, 2018; Zhang et al., 2018) play important roles in the regulation of cellular (e.g., mitochondrial) metabolic activity. Given that breathing levels are modulated to assure that metabolic demands are met, our future studies must determine the potential changes in metabolic activity at rest and during HX challenge in HDAC6 KO mice to determine how these changes relate to the ventilatory parameters observed in the present study.

## Conclusion

The present study demonstrates that the loss of HDAC6 in male C57BL/6 mice has a profound impact on the expression of ventilatory responses to a HX challenge. This study raises the possibility that pharmacological blockade of HDAC6 (Godena et al., 2014; Majid et al., 2015; Cuadrado-Tejedor et al., 2017; Esteves et al., 2018) may provide deeper insights into the roles of HDAC6 in the control of breathing in healthy and disease states. The genetic bases for different breathing patterns in different strains of mice at rest and in response to hypoxic and hypercapnic challenges have received extensive study (Tankersley et al., 1994; Tankersley et al., 1998; Tankersley et al., 2000a; Tankersley et al., 2000b; Tankersley et al., 2000c; Han and Strohl, 2000; Tankersley, 2000; Tankersley, 2001; Tankersley, 2003; Tankersley and Broman, 2004; Balbir et al., 2006; Gillombardo et al., 2012; Strohl et al., 2012) as have the genetic bases for the differences involve many neurochemical processes (Tankersley et al., 2002a; Tankersley et al., 2002b; Tankersley et al., 2002c; Price et al., 2003; Groeben et al., 2005; Yamauchi et al., 2008a; Yamauchi et al., 2008b; Moore et al., 2012; Moore et al., 2014), and structural features of respiratory structures, such as the carotid body (Yamaguchi et al., 2003; Yamaguchi et al., 2006; Chai et al., 2011). The possibility that HDAC6 is a key player in the genetic factors that regulate ventilatory control processes *per se*, and those that respond to a hypoxic gas challenge, opens up intriguing avenues of research including those testing whether administration of selective HDAC6 inhibitors, such as CAY10603, Tubacin and Nexturastat (Lu et al., 2017; Govers et al., 2019; Ma et al., 2019; Sanaei and Kavoosi, 2019; Sun et al., 2019), augment/stabilize ventilatory responses to hypoxic and/or hypercapnic challenges in mouse models, such as C57BL/6 mice (Yamauchi et al., 2007; Yamauchi et al., 2008a; Yamauchi et al., 2008b; Yamauchi et al., 2008c; Yamauchi et al., 2010).

## Data Availability

The raw data supporting the conclusions of this article will be made available by the authors, without undue reservation.
