# Supplementary material for: Male histone deacetylase 6 (HDAC6) knockout mice have enhanced ventilatory responses to hypoxic challenge
Source: Front Physiol. 2024 Feb 6;14:1332810. doi: 10.3389/fphys.2023.1332810 (PMC10880035; doi:10.3389/fphys.2023.1332810)
Supplement: Supplementary file 1 [file Table1.docx]

**Supplementary Material**

**Male histone deacetylase 6 (HDAC6) knockout mice have enhanced ventilatory responses to hypoxic challenge**

Paulina M. Getsy^a^

Gregory A. Coffee^a^

Thomas J. Kelley^a,b^

Stephen J. Lewis^a,c,d^

*^a^Department of Pediatrics, Case Western Reserve University, Cleveland, OH 44106, USA*

*^b^Department of Genetics and Genome Sciences, Case Western Reserve University, Cleveland, OH 44106, USA*

*^c^Department of Pharmacology, Case Western Reserve University, Cleveland, OH 44106, USA*

*^d^Functional Electrical Stimulation Center, Case Western Reserve University, Cleveland, OH 44106, USA*

***Address correspondence to:** Stephen J. Lewis, PhD. Department of Pediatrics, Case Western Reserve University, 10900 Euclid Avenue, Cleveland, OH 44106-4984, USA. Telephone: 843-422-7639. Email: sjl78@case.edu

**Supplementary Figure S1**

**Supplementary Figure S1.** A gel Polymerase Chain Reaction (PCR) for genotyping HDAC6 mice. Genotyping was completed by PCR analysis using DNA extracts from ear biopsies of the male mice. To detect the HDAC6 knockout allele (250 bp) primers (5’-CCATGACCGAGATCGGCGAGCA-3’) and (5’-CGTGAATTCCGATCATATTCAAT -3’) were used. To detect the HDAC6 wildtype allele (350 bp) primers (5’- CTGGTTCGTCTGAAGACA -3’) and (5’- GTGGACCAGTTAGAAGCC -3’) were used. PCR reactions were completed for 30 cycles of 95°C for 30 sec, 55°C for 30 sec and 72°C for 90 sec.

**Supplementary Table S1**

Definition of ventilatory parameters used in this study

| **Parameter** | **Abbreviation** | | **Units** | | **Definition** |
| --- | --- | --- | --- | --- | --- |
| **A. Directly recorded parameters** | | | | | |
| Frequency of breaths | | Freq | | breaths/min | Rate of breathing |
| Inspiratory Time | | Ti | | sec | Duration of inspiration |
| Expiratory Time | | Te | | sec | Duration of expiration |
| End Inspiratory Pause | | EIP | | msec | Pause between end of inspiration start of expiration |
| End Expiratory Pause | | EEP | | msec | Pause between end of expiration and start of inspiration |
| Relaxation time | | RT | | sec | Decay of expiration to 36% maximum |
| Tidal Volume | | TV | | mL | Volume of inspired air per breath |
| Peak Inspiratory Flow | | PIF | | mL/sec | Maximum inspiratory flow |
| Peak Expiratory Flow | | PEF | | mL/sec | Maximum expiratory flow |
| Expiratory flow at 50% | | EF_50_ | | mL/sec | Expiratory flow at 50% expired TV |
| Non-eupneic breathing index | | NEBI | | % | % of non-eupneic breaths per epoch |
| **B. Derived parameters** | | | | | |
| Minute Ventilation | | Freq x TV | | mL/min | Total volume of air inspired per min |
| Ti/Te | | Ti/Te | | none | Inspiratory quotient |
| PEF/PIF | | PEF/PIF | | none | Flow balance |
| Expiratory Delay | | Te-RT | | No units | Measure of elongated expiration |
| Inspiratory Drive | | TV/Ti | | mL/sec | Central urge to inhale |
| Expiratory Drive | | TV/Te | | mL/sec | Central drive to exhale |
| NEBI/Frequency | | NEBI/Freq | | %/(breaths/min) | Balanced rejection index |

**Supplementary Figure S2**


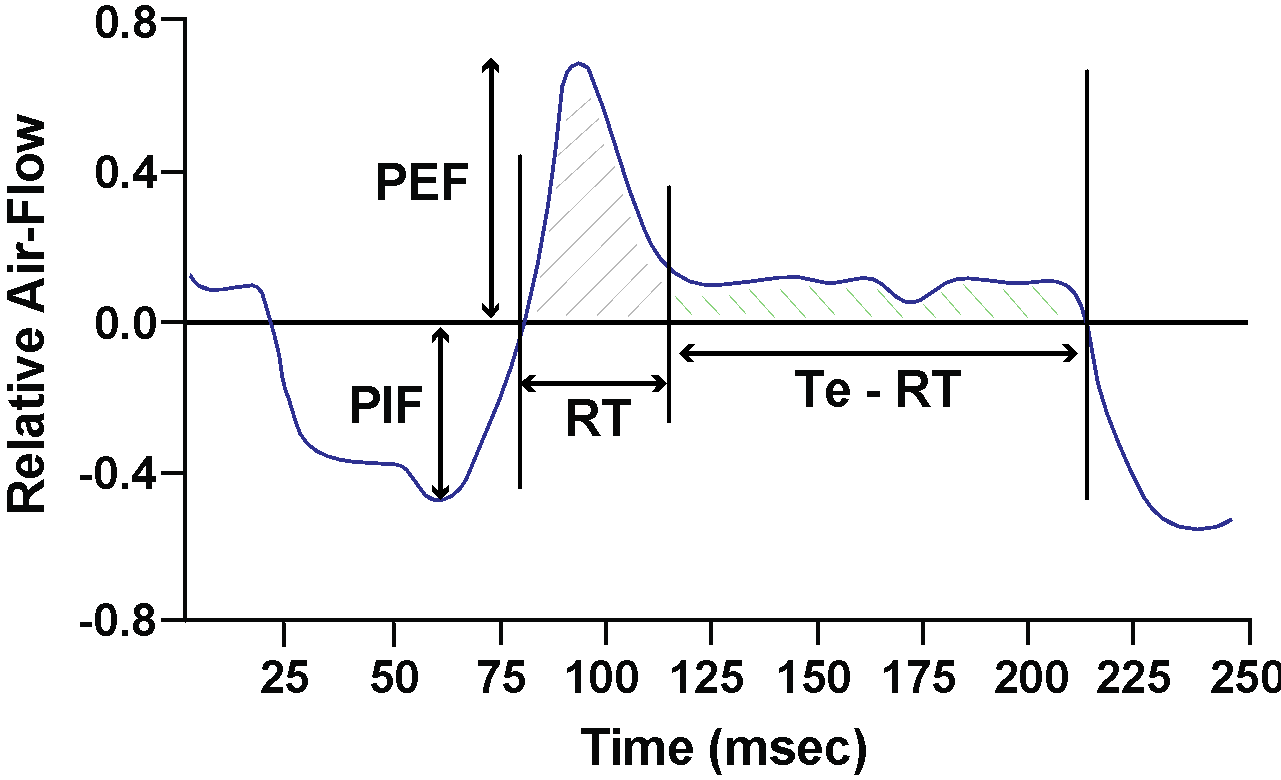


**Supplementary Figure S2.** Relationship between peak inspiratory flow (PIF), peak expiratory flow (PEF), relaxation time (RT) and expiratory time (Te).
